# A Prospective Clinical Audit to Improve Compliance With Perioperative Atrial Fibrillation Prevention Protocols in Off-Pump Coronary Artery Bypass Surgery

**DOI:** 10.7759/cureus.103090

**Published:** 2026-02-06

**Authors:** Md. Ziaur Rahman, Mushfiqun Islam, Md. Sajidujjaman, Snahashish Chakroborty, SM Rashidul Hasan

**Affiliations:** 1 Department of Cardiac Surgery, National Heart Foundation Hospital and Research Institute, Dhaka, BGD; 2 Department of Cardiothoracic Surgery, Apollo Imperial Hospitals, Chittagong, BGD; 3 Department of Cardiac Anesthesia, Apollo Imperial Hospitals, Chittagong, BGD

**Keywords:** atrial fibrillation, beta-blocker, cardiac surgery, off-pump coronary artery bypass, potassium management, quality improvement

## Abstract

Background

New-onset atrial fibrillation (AF) is a frequent complication following cardiac surgery and is associated with significant mortality and morbidity. This audit evaluated adherence to perioperative AF prevention standards and the effect of targeted quality-improvement measures in off-pump coronary artery bypass grafting (OPCAB) patients.

Methodology

Two prospective audit cycles were performed at the Apollo Imperial Hospitals. Cycle 1 (January to September 2024; n=95 eligible) established baseline compliance with three standards: the continuation of beta-blocker on the morning of surgery, maintenance of perioperative serum potassium at 4.5-5.0 mmol/L, and reinstitution of beta-blocker within 12 hours postoperatively. After multidisciplinary interventions (permanent ICU/OR posters, checklist attached to patient records, weekly team meetings, and four-times-daily ward rounds), improvement was assessed in cycle 2 (November 2024 to July 2025; n=101). Bisoprolol was the institutional beta-blocker; ivabradine was used if beta-blockers were contraindicated. Categorical variables were analyzed using the chi-square test or Fisher’s exact test, and relative risk (RR) and odds ratios (OR) were estimated with 95% confidence intervals (CI).

Results

Preoperative beta-blocker continuation improved from 66.3% to 94.1% (p<0.001). Serum potassium maintenance within target increased from 42.1% to 81.2% (p<0.001). Reinstitution rates were high in both cycles (96.8% versus 95.0%, p=0.72). New-onset AF incidence decreased from 34.7% to 16.8% (p=0.0067; RR: 0.48 {95% CI: 0.29-0.81}; OR: 0.38 {95% CI: 0.19-0.74}).

Conclusion

Structured, multidisciplinary quality-improvement measures significantly improved compliance with AF prevention protocols and were associated with a decrease in postoperative AF incidence by 50% in OPCAB patients. Routine audits and sustained adherence to protocol are recommended.

## Introduction

Postoperative atrial fibrillation (AF) is one of the most frequent complications following cardiac surgery, affecting approximately 30%-50% of patients, and is associated with increased stroke, length of hospital stay, and mortality [1,2]. The guidelines recommend the use of prophylactic beta-blockers for the prevention of AF and the targeted correction of electrolyte disturbances to reduce the risk of arrhythmia [3]. The present audit aimed to assess baseline compliance with selected perioperative AF prevention standards in patients undergoing off-pump coronary artery bypass grafting (OPCAB), implement targeted interventions, and measure the effect on compliance and the incidence of AF.

## Materials and methods

This is a prospective clinical audit with two cycles conducted in the Department of Cardiothoracic and Vascular Surgery, the Apollo Imperial Hospitals, Chittagong, Bangladesh. The audit and reaudit were conducted on consecutive adult patients undergoing isolated off-pump coronary artery bypass grafting (OPCAB).

Inclusion criteria

All consecutive patients aged 18-75 undergoing OPCAB were included in this audit.

Exclusion criteria

Patients with an age of >75 years, preoperative atrial fibrillation, redo coronary artery bypass grafting (CABG), on-pump CABG, combined valve and CABG procedures, severe left ventricular dysfunction (as defined by ejection fraction {EF} of <30%), and heart block were excluded from this audit.

The authors’ perioperative atrial fibrillation prevention protocol consisted of continuing beta-blocker therapy in the preoperative period, initiating beta-blockers early in the postoperative phase (within 12 hours of inotrope tapering), and maintaining serum potassium levels between 4.5 and 5.0 mmol/L [4]. These measures constituted the audit standards, as outlined in Table 1.

**Table 1:** Audit standards

Standard	Target (%)	Exceptions
Continuation of beta-blocker therapy on the day of surgery	100%	Bradycardia, hypotension, and heart block
Perioperative serum potassium maintained within 4.5-5.0 mmol/L	80%	None
Reinstitution of beta-blockers within 12 hours post-inotropes	100%	Bradycardia, hypotension, and heart block

**Figure 1:**
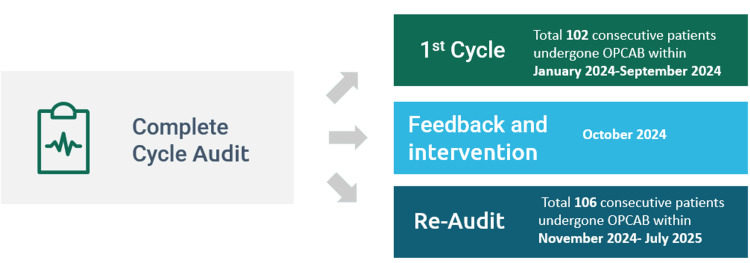
Patient enrollment in both audit cycles OPCAB: off-pump coronary artery bypass grafting

Patient enrollment in both audit cycles was determined after applying the predefined inclusion and exclusion criteria, as illustrated in Figure 1.

Study procedure

**Figure 2:**
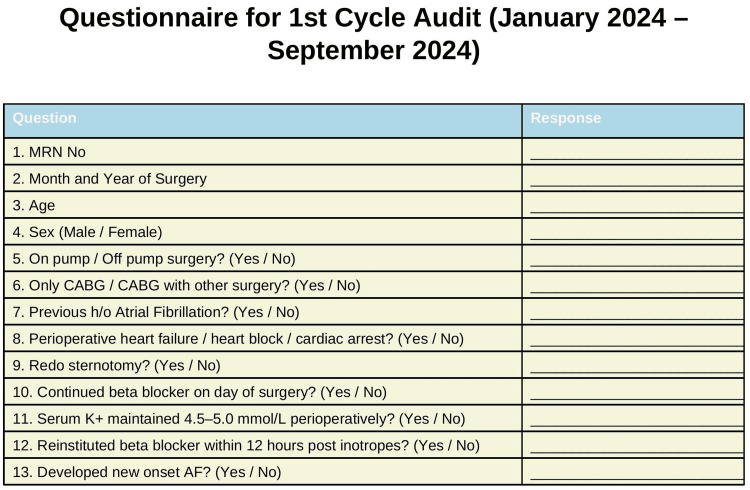
Audit questionnaire on the first cycle of data collection MRN, medical record number; CABG, coronary artery bypass grafting; AF, atrial fibrillation; h/o, history of

Oral bisoprolol was the choice beta-blocker. Oral ivabradine was used when beta-blockers were contraindicated. Demographics and compliance data were prospectively recorded in the audit questionnaire. Continuous four-lead ECG monitoring in the cardiac ICU and routine serum potassium measurements were done once preoperatively, 2-3 times intraoperatively, every four hours on the “zero” postoperative day (POD), every six hours on the first POD, every eight hours on the second POD, and every 12 hours in the 3rd-5th POD from arterial and venous blood gas analysis. The data from the patients in the first cycle were collected in a questionnaire, which is illustrated in Figure 2.

**Figure 3:**
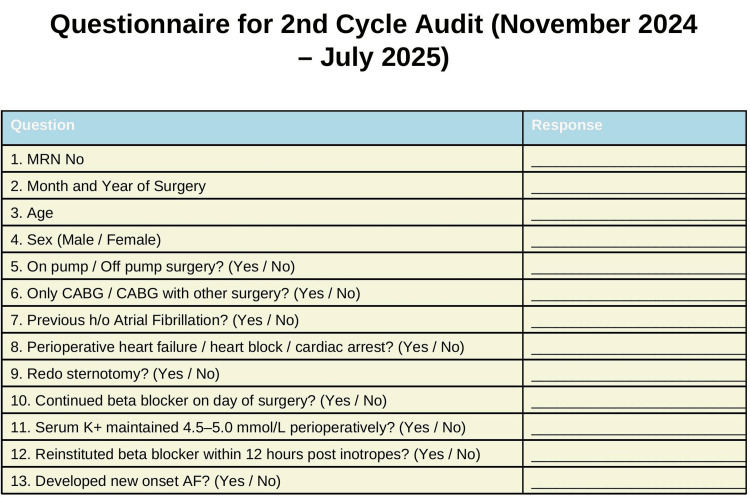
Audit questionnaire on the second cycle of data collection MRN, medical record number; CABG, coronary artery bypass grafting; AF, atrial fibrillation; h/o, history of

The results of the first-cycle audit were discussed in team meetings and ward rounds following data collection in the first cycle, with an emphasis on improving compliance to meet standards. Permanent posters in the ICU, OR, and doctors’ and nurses’ stations were displayed, emphasizing the standards. A structured checklist was attached to patient records, and weekly multidisciplinary meetings (surgeons, anesthetists, and nurses) were held. Additionally, ward rounds were conducted four times daily. All interventions were uniformly applied to patients in cycle 2 (reaudit). After the interventions, compliance with each standard and the incidence of new-onset AF were measured again and recorded in the audit questionnaire, which is the second cycle of the audit/reaudit, as illustrated in Figure 3.

The audit protocol was approved by the Institutional Review Board (IRB) of the Apollo Imperial Hospitals, Chattogram, Bangladesh (approval number: AIHL/IRB/25092), and a waiver of informed consent was granted according to the institutional protocols for clinical audits.

Statistical analysis

Categorical variables are presented as counts and percentages. Between-cycle comparisons used chi-square or Fisher’s exact tests as appropriate. Relative risk (RR) and odds ratio (OR) were calculated with 95% confidence intervals (CI). A two-sided p<0.05 was considered significant. Data were collected and compiled using Microsoft® Excel® 2016 Microsoft Office (MSO) (version 2408, build 16.0.17932.20620, 64-bit; Microsoft Corp., Redmond, WA) and were analyzed with IBM SPSS Statistics for Windows (version 23.0, released 2015; IBM Corp., Armonk, NY).

## Results

The initial audit (cycle 1) included 102 consecutive patients. Seven were excluded, leaving 95 patients eligible for analysis. The reaudit (cycle 2) included 106 consecutive patients; five were excluded, leaving 101 patients eligible for the reaudit. Patient demographics are summarized in Table 2.

**Table 2:** Demographic data SD: standard deviation

Characteristics	Cycle 1 (n=95)	Cycle 2 (n=101)
Mean age, years (±SD)	56.03±8.89	55.65±9.12
Male, n (%)	88 (92.6%)	89 (88.1%)
Female, n (%)	7 (7.4%)	12 (11.9%)
Mean age, men (±SD)	56.31±8.76	55.85±8.97
Mean age, women (±SD)	52.29±10.58	54.59±10.07
Excluded (n)	7	5

Key findings

In the first cycle, 63 of 95 patients received a beta-blocker on the morning of their surgery (66.3%). During the reaudit after the intervention, 95 of 101 patients (94.1%) received morning beta-blockers on the day of their surgery (p<0.001). In the first cycle, 40 of 95 patients (42.1%) were able to maintain their serum K^+^ within the range of 4.5-5.0 mmol/L. After intervention and reaudit, we found that 82 of 101 patients (81.2%) were able to maintain serum K^+^ within the range (p<0.001). Ninety-two out of 95 patients (96.8%) were reinstituted on beta-blocker within 12 hours after weaning off the inotropes during the first cycle, while 96 out of 101 patients (95.0%) were reinstituted on beta-blocker within 12 hours post-inotropes during the second cycle after intervention (p=0.72). A comparison of compliance in cycle 1 and cycle 2 is summarized in Table 3.

**Table 3:** Comparison of compliances in cycle 1 and cycle 2

Standard	Cycle 1 (n=95)	Cycle 1 (%)	Cycle 2 (n=101)	Cycle 2 (%)	P-value
Morning beta-blocker ensured in patients on the day of surgery	63	66.3%	95	94.1%	<0.001
Perioperative serum K^+^ maintained within 4.5-5.0 mmol/L	40	42.1%	82	81.2%	<0.001
Reinstitution of beta-blocker within 12 hours after weaning of inotropes	92	96.8%	96	95.0%	0.72

Outcome

Thirty-three out of 95 patients (34.7%) developed atrial fibrillation during the initial audit. After intervention and reaudit, atrial fibrillation developed in 17 of 101 patients (16.8%) (p=0.0067). The relative risk was 0.48 (95% confidence interval: 0.29-0.81), and the odds ratio was 0.38 (95% confidence interval: 0.19-0.74). A comparison of new-onset perioperative AF development between the two cycles is summarized in Table 4.

**Table 4:** Comparison of new-onset perioperative AF development between the two cycles RR, relative risk; OR, odds ratio; AF, atrial fibrillation; CI, confidence interval

Outcome	Cycle 1 (n=95)	Cycle 2 (n=101)	RR (95% CI)	OR (95% CI)	P-value
New-onset AF	33 (34.7%)	17 (16.8%)	0.48 (0.29-0.81)	0.38 (0.19-0.74)	0.0067

## Discussion

**Figure 4:**
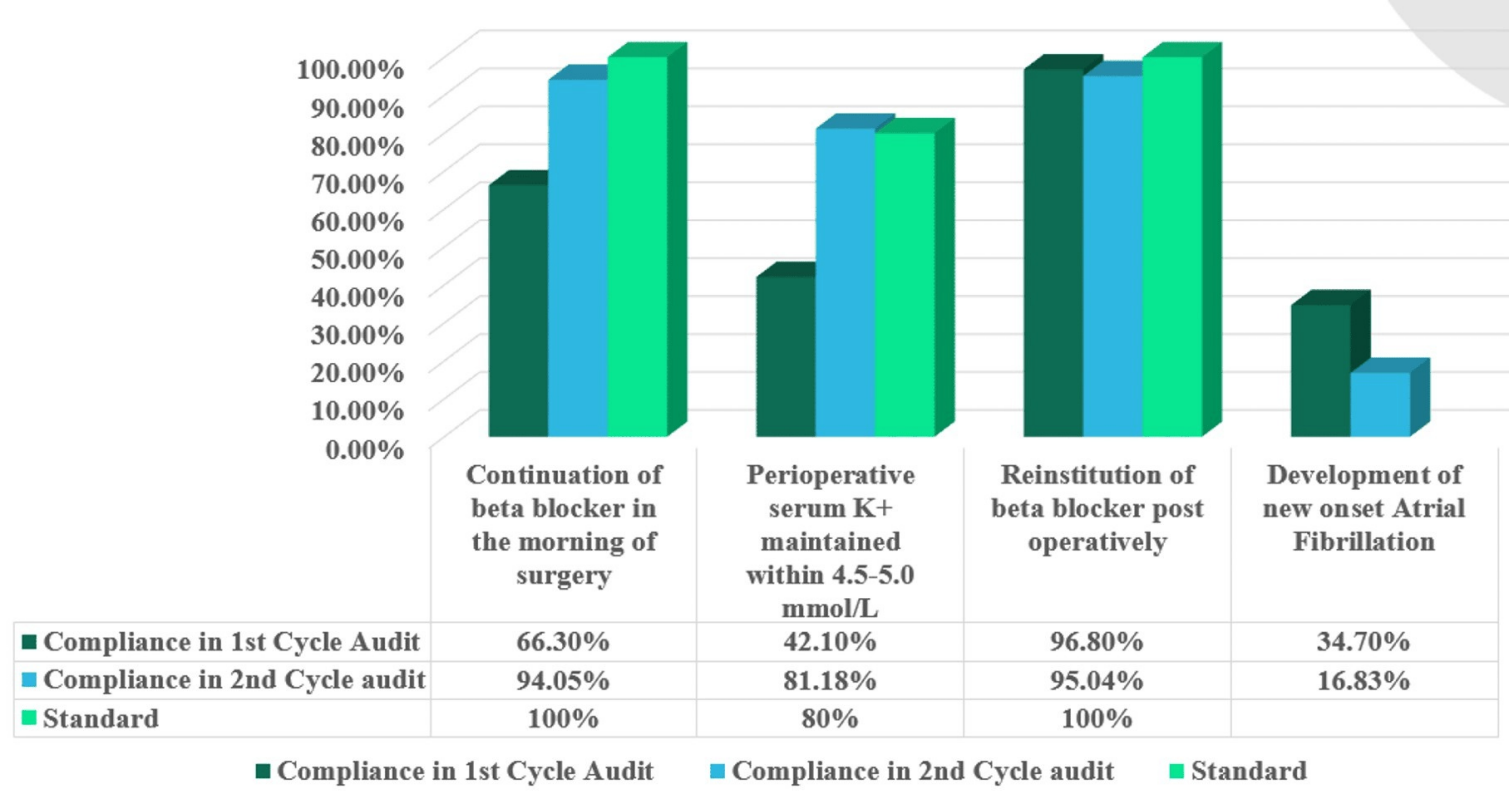
Overall comparison of audit standards in both cycles and the incidence of AF AF: atrial fibrillation

This prospective audit demonstrated that targeted, cost-efficient quality-improvement initiatives substantially improved adherence to perioperative atrial fibrillation preventive protocols and were associated with a significant reduction in new-onset atrial fibrillation following off-pump coronary artery bypass surgery. The initial incidence of AF (34.7%) in cycle 1 aligns with established ranges in cardiac surgery groups, and the subsequent reduction to 16.8% after the intervention indicates a clinically meaningful improvement [5,6]. The continuation of beta-blockers before surgery and their immediate resumption are crucial procedures supported by guidelines and meta-analyses demonstrating that prophylactic beta-blockade reduces the incidence of postoperative atrial fibrillation (POAF) [3-6]. In both observational and interventional studies, the effective management of electrolytes, particularly maintaining serum potassium levels within the upper-normal range, has been associated with a decreased occurrence of postoperative atrial fibrillation [5-8]. The combined efforts of maintaining medication compliance, carefully controlling potassium levels, and patient-oriented team efforts likely contributed to a decrease in the number of atrial fibrillation cases. The general comparison of compliance with the requirements in both cycles, as well as the downfall of new-onset atrial fibrillation, is illustrated in Figure 4.

The reported incidence of new-onset AF after cardiac surgery is 10%-40% [9]. Previous studies have demonstrated that preoperative β-blocker continuation is a Class I recommendation to prevent AF, and omission increases the risk of postoperative AF [10]. SCA/EACTA guidelines note 10%-90% continuation rates [11]. At our center, 94.1% of CABG patients received β-blockers preoperatively.

SCA/EACTA guidelines recommend immediate postoperative β-blockers (within 24 hours) as a Class I, Level A recommendation to counter sympathetic activation triggering AF. Adherence varied from 10% to 90% [11]. Buerge et al. found that early β-blocker initiation/reinitiation post-cardiac surgery correlated with lower new-onset AF and rates increasing from 82.7% to 91.3% after care bundle adoption [12]. Our institute mandates β-blockers within 12 hours of tapering inotropes, followed in 96.8% of initial audit (cycle 1) and 95% of reaudit (cycle 2).

Hypokalemia contributes to ventricular/supraventricular arrhythmias, but Lancaster et al. found that potassium supplementation does not guarantee protection from AF, indicating weak evidence [13]. However, our protocol mandates perioperative serum K⁺ level to be kept within 4.5-5.0 mmol/L. Of the initial audit, 42.1% met this, with corrections for deviations. We reinforced potassium maintenance training, especially in patients with diuretic use, and the reaudit ensured 81.2% compliance.

Apart from these three factors/standards in this audit, one study showed that the reduced incidence of new-onset AF in post-CABG patients may be due to the off-pump nature of the surgery [14]. Again, another study shows that there is no association between on-pump surgery and an increased incidence of AF [15,16].

Reinstitution rates were already higher in cycle 1, which made it less likely that improvements would happen. The most significant changes were observed in the continuation of morning beta-blockers and potassium management, which aligned with the areas on which instructional programs and checklists focused. The strengths of this audit are its prospective design, sequential sampling, and the implementation of system-level interventions with measurable effects. The audit did not aim to determine causality; however, the temporal correlation and biological plausibility support the existence of a true impact.

To authenticate outcomes, future efforts should focus on maintaining existing gains, exploring the use of automatic electronic reminders for medication reconciliation, and considering multicenter collaboration. Randomized studies could evaluate adjunctive medications, such as ivabradine, for patients with contraindications to β-blockers.

Limitations

Atrial fibrillation (AF) can occur after off-pump coronary artery bypass grafting (CABG) due to a number of factors, such as being older and having chronic obstructive pulmonary disease (COPD), congestive heart failure, poor left ventricular function, and myocardial ischemia [4-9]. Nevertheless, because this study was a clinical audit, it did not evaluate causal relationships between the identified factors and postoperative atrial fibrillation. Rigorous case-control studies are required to substantiate these associations.

## Conclusions

Structured interdisciplinary quality-improvement initiatives significantly improved compliance with perioperative atrial fibrillation preventative strategies and were associated with a 50% reduction in new-onset atrial fibrillation among patients undergoing off-pump coronary artery bypass grafting. The findings highlight that relatively simple, low-cost system-based interventions can translate into meaningful clinical benefits when consistently applied. Importantly, the improvement observed was driven by the enhanced reliability of routine perioperative practices rather than the introduction of new pharmacological therapies. This underscores the value of process optimization in high-risk surgical pathways. Regular audits, continuous feedback, and sustained team engagement should therefore be considered integral components of contemporary cardiac surgical care to ensure durable improvements in patient outcomes.
